# Adapting the CIERA framework to assess road infrastructure resilience to climate-related events

**DOI:** 10.4102/jamba.v17i2.1844

**Published:** 2025-07-10

**Authors:** Zaheer Doomah

**Affiliations:** 1 Department of Civil Engineering, Faculty of Engineering, University of Mauritius, Reduit, Mauritius

**Keywords:** resilience, CIERA, road infrastructure, robustness, recoverability, adaptability

## Abstract

Roads form part of the essential physical infrastructure but face numerous external threats throughout their lifespan ranging from physical, meteorological and even operational threats. With climate change, roads are becoming increasingly vulnerable to adverse events, with an urgent need for building resilience in this type of critical infrastructure. This study focused on the adaptation of the Critical Infrastructure Elements Resilience Assessment (CIERA) method for measuring the resilience of road infrastructure with respect to climate-related events. A qualitative approach was adopted to identify the parameters to be measured under the three main components of the CIERA framework, namely robustness, recoverability and adaptability. Semi-structured interviews were carried out with a purposive sample of 15 experts in the transportation field working in both the public and private sector. This yielded the various indicators of road infrastructure resilience for inclusion in the CIERA framework.

**Contribution:**

The study identified 32 indicators to be assessed for road infrastructure resilience. The most cited ones for the robustness component include the implementation of protective security measures, adoption of new design standards and availability of alternative routes, whereas for the recoverability component, fund allocation, pre-approved response plans and agreements with third parties for help during disasters have been most highlighted by interviewees. Lastly, appropriate risk management practices, investment in technological innovation and provision of training are considered important aspects for the adaptability component. The framework can be applied in the road transportation sector to assess the level of resilience and guide decisions at strategic levels for investment.

## Introduction

Critical infrastructure (CI) is a set of systems essential for providing services and goods that are crucial to the functioning of the national economy and the livelihood of people (Quitana, Molinos-Senante & Chamorro 2020). They include power grids, transportation systems, telecommunication systems and water supply networks, and their proper functioning depends on each other because of their interconnectedness (Liu, Fang & Zio 2021). Road transport infrastructure consisting of roads, highways, bridges and other associated installations (Splichalova & Flynnova 2021) serve the society in various aspects of daily activities. However, the transport infrastructure is vulnerable to disruption because of internal and external threats, which can severely affect their operation (Kermanshah & Derrible 2016). It is therefore essential to understand how to improve land transport systems’ resilience to ensure that these disruptions are prevented or short-lived and the infrastructure maintains a high level of reliability.

Resilience of road transport infrastructure refers to the capacity of a system or element to absorb, adapt to and quickly recover from potentially disruptive events and is an essential characteristic for protecting CI (Splichalova et al. 2020). During the past decade, several climatic events such as floods, earthquakes and storms have significantly impacted road infrastructure with an adverse effect on human livelihood and the economy (Fang & Zio 2019; Zhang & Alipour 2022). Against this backdrop of climate emergency, the creation of disaster-resilient CI and societies is becoming essential. To achieve this aim, developing assessment methods to determine the capacities and performance of these systems is a key aspect. These frameworks are often based on indicators, which allow measuring various characteristics of the CI elements and assessing their performance. This helps to evaluate the capacity of these elements when under disruption and provide information to stakeholders for decision-making (Yang et al. 2022). Several methods have been developed over the past decade for the measurement of resilience in transportation infrastructure, but these focused mostly on system-based analysis using mathematical modelling and simulation (Nickdoost et al. 2024; Tan et al. 2022; Tang et al. 2020; Zang et al. 2024). Studies adopting a conceptual approach and survey-based methodology have been found to be less prevalent (Wan et al. 2017), while research on the classification of indicators to allow better application in case studies is also lacking (Yang et al. 2022).

The challenge with incorporating resilience lies with agencies being able to assess the vulnerabilities of their infrastructure and take measures to ensure that both existing and future elements of the system are resistant to disruptions. In line with this, it was proposed that studying resilience at element level instead of network level would help to identify weak points and take mitigation measures to protect these elements against failure and thus increase resilience levels (Rehak et al. 2019). Based on this, the Critical Infrastructure Elements Resilience Assessment (CIERA) framework was developed and will be further described in the next section. Specific indicators were then developed subsequently for CI elements in the electricity sector (Rehak et al. 2019), the emergency services sector (Splichalova et al. 2020) and the energy sector (Rehak et al. 2023). The same approach can be applied in the road transportation sector to develop a set of indicators that can reliably assess the level of resilience at both technical and organisational levels, understand the vulnerabilities of specific elements and guide decisions at strategic levels.

Therefore, this study aimed to address the voids in the existing literature by developing a set of indicators for the road infrastructure sector based on the CIERA methodology using a conceptual approach through review of existing studies and interviews of transportation sector professionals.

## Critical infrastructure elements resilience assessment framework

The term ‘resilience’ was first used in the ecology domain to describe how much stress the system can withstand while keeping its integrity (Holling 1973). Over the past decades, the resilience concept has been applied in various fields with differing definitions. However, there appears to be a consensus on the recurring dimensions of a resilient system. Most definitions (Bruneau et al. 2003; Fang & Zio 2019; Liu et al. 2021; Serre & Heinzlef 2018) invoke the ability of a system to:

[*R*]eturn to normal quickly, avoiding damage or permanent change as a result of disturbance, with the level of resilience measured by the time it takes for a system to move back to some specified degree of its previous state.

Resilience assessment frameworks have thus been developed for infrastructure systems, whose resilience refers to their ability to absorb, resist, adapt to and recover from the effects caused by a disruptive event (Osei-Kyei et al. 2021). Bruneau et al. (2003) developed a conceptual framework for the measurement of resilience with four main properties: (1) robustness: strength or the ability of elements or systems to withstand a stress level without reduction or loss of function; (2) redundancy: the degree to which elements or systems can be replaced by alternative pathways; (3) resourcefulness: the ability, during disruptive events, to identify problems, establish priorities and mobilise resources; and (4) rapidity: the capability to achieve set targets timely to mitigate losses and reduce future disturbances. On the other hand, Chester and Allenby (2019) argued that, for improved resilience, transportation systems should not only have the adaptive capacity to respond to threats and changes by being modular and connected but should also be supported by a competent and organic organisational culture that promotes flexibility and agility through cooperation, people empowerment, innovation and decentralised decision-making processes. These are in line with resilience traits in socio-ecological systems that include flexibility, modularity, diversity, openness to learning, innovation and transformation (Shakou et al. 2019).

The CIERA framework was developed by Rehak et al. (2019) based on the principle that a system’s performance is highly dependent on its weakest points and identifying these would help to better allocate available resources to improve resilience. It comprises two variables pertaining to the technical dimension of resilience, namely robustness and recoverability, and one variable related to organisational resilience in terms of adaptability. These dimensions have been further broken down into 12 variables and are applicable to various types of CI system as shown in Table 1.

**TABLE 1:** Critical infrastructure elements resilience assessment framework components and associated variables.

Components	Variables	Definitions
**Robustness** *Ability of an element to absorb the impacts of a disruptive event*	Crisis preparedness	Set of planning documents to increase readiness for disruptive events and implementation of security measures
	Redundancy	Capacity to replace a disturbed part of an element without affecting performance
	Detection capability	Time needed or probability of foreseeing a disruptive event
	Responsiveness	Probability or time of intervention to mitigate the impacts of a disruptive event
	Physical resistance	Capacity to withstand the impacts of a disruptive event
**Recoverability** *Capacity of an element to recover its function to the required level after a disruptive event*	Material resources	Availability of components for the repair or replacement of parts affected by disruptive events
	Financial resources	Availability of funds and resources to finance quick restoration of element affected during disruptive event
	Human resources	Availability of people with the required level of knowledge
	Recovery processes	Processes that support quick recovery of an affected element’s performance
**Adaptability** *Ability of an operator to prepare an element for the potential effects of disruptive events*	Risk management	Processes supporting early risk assessment and management such as analysis of various scenarios for disruptive events
	Innovation processes	Measures that support research and transformation in terms of security measures and organisation structure
	Education and development processes	Processes supporting the knowledge, skills and attitudes of employees in critical infrastructure organisations

*Source*: Adapted from Rehak, D., Senovsky, P., Hromada, M. & Lovecek, T., 2019, ‘Complex approach to assessing resilience of critical infrastructure elements’, *International Journal of Critical Infrastructure Protection* 25, 125–138. https://doi.org/10.1016/j.ijcip.2019.03.003

However, the key indicators for the 12 variables for inclusion in the CIERA framework have not yet been investigated for the measurement of road infrastructure resilience to climate-related events and have been the focus of this study.

## Research methods and design

### Study setting

**FIGURE 1:**
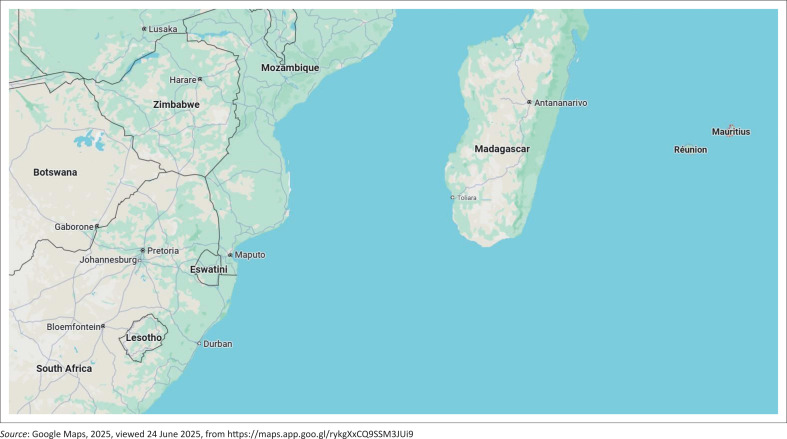
Map showing location of Mauritius in the Indian Ocean.

Mauritius is a Small Island Developing State located in the Indian Ocean, off the eastern coast of Africa, as shown in Figure 1.

Although a light transit system has recently been implemented in the island to link the main towns and modernise the transport landscape, several major road projects are being implemented to provide improved accessibility and mobility while catering for the increasing fleet of vehicles. The land transportation sector is essential for the economic activities in Mauritius, and this is similarly the case for African countries where there is significant investment in transport infrastructure to cater for increasing urbanisation and economic pressures (Mtweve et al. 2025). However, recent adverse climatic events such as cyclones and flash floods have shown that the road infrastructure is highly vulnerable to flooding (Luiu et al. 2025), and there is a pressing need to build climate resilience in road planning and development (Mashamaite 2025). According to the World Risk Report of 2024, like most countries in the African continent, Mauritius still lags behind in terms of adaptive capacities to climate change events. With an estimated one in four people likely to be directly exposed to a 100-year flood event in the future with global warming (Rentschler, Salhab & Jafino 2022), there is therefore an urgent requirement to improve resilience of the land transport system in African countries to reduce the economic impacts and ensure that communities are not cut off from essential services during flood events.

### Study design

A qualitative research design was adopted for this study, as it allows for exploring and understanding complex situations and inductively captures meanings from individuals’ personal experiences on specific issues (Creswell & Creswell 2018, Saldana 2011). This approach was more appropriate than the quantitative one at this stage because of the absence of pre-determined indicators. Thus, the exploratory design allows researchers to answer the research question set by obtaining direct quotations from persons based on their lived experiences and knowledge, using open-ended questions that lead to emerging options (Patton 2005). This interpretive inquiry, which focuses on obtaining new indicators for assessing resilience of road infrastructure, is therefore well suited as it places emphasis on the viewpoints of road sector experts. Two stages were adopted for the study: (1) an extensive literature review; and (2) semi-structured interviews with key transportation experts working in the road infrastructure sector.

The initial comprehensive literature review was essential to gain an insight on the topic and allowed the preliminary identification of indicators for the assessment of resilience for inclusion in the CIERA framework. Some studies considered infrastructure systems in the water sector (Dalmond 2015), energy sector (Rehak et al. 2023) and emergency rescue (Splichalova et al. 2020), while others focused on transport systems (Besinovic 2020; Dvořák & Hoterová 2022). In the second stage, semi-structured interviews with transportation sector experts helped to capture important information through discussion and interaction, while also obtaining contacts of other potential participants. The study used a grounded theory approach to identify indicators with the procedures for thematic analysis developed by Braun and Clarke (2006). The analysis consisted of the following main steps: (1) collecting data through semi-structured interviews; (2) transcription, familiarisation with the data and selection of quotations; (3) identification of keywords; (4) coding; (5) theme generation to identify indicators; and (6) producing a figure that depicts the conceptual model emerging from the data analysis.

This approach allowed to identify indicators pertaining to the road infrastructure, with several similar to previous studies but some more specific to the road sector.

### Sampling strategy

Sampling for grounded theory is more focused on obtaining participants with relevant experience related to the topic of study, with data collection aimed at reaching data saturation. A non-probability purposive sampling coupled with snowballing technique was therefore used to select 15 participants working in the road transportation sector in Mauritius as engineers, project managers and policymakers. The participants have been actively involved in the planning, design, construction and maintenance of road infrastructure in Mauritius and are therefore well aware of the issues faced during disruptive events. The experts in the client organisations are also knowledgeable on the key technical and organisational issues faced during climatic events at both management and policy levels as they participate in activities during and after disturbances to restore service levels. The selection criterion for participants was having at least 5 years of work experience in the sector. The participants included six experts from three client organisations and nine from six local consultancy firms involved in road infrastructure projects. Data saturation was obtained on the 12th interview, with three more conducted to ensure and confirm that no new insight was being obtained. This is in line with previous studies, which found that for thematic analysis, data saturation is usually reached with a sample of 12 to 20 participants (Ahmed 2025; Guest, Bunce & Johnson 2006).

### Data collection and analysis

Interviewing is a commonly used method for collecting data and can be classified as being ‘structured’, ‘semi-structured’ or ‘unstructured’ depending on the degree of flexibility in the questions and answers format (Denscombe 2021). This study adopted the semi-structured interview format, with interviews conducted in-person at the offices of the participants after having sought appointments. Data capture was performed through note taking because of the reluctance of participants to be audio-recorded. This step allowed all interviewees to be asked the same questions but within a flexible framework (Dearnley 2005) and aimed at identifying the resilience indicators for road infrastructure with respect to climate-related threats. Data collected were then transcribed, analysed using the thematic analysis approach and triangulated with the literature review findings to obtain a refined list of resilience indicators.

### Ethical considerations

The study consisted of obtaining insights from participants based on their experience in the road transportation sector. Data were collected through one-time face-to-face interviews, and all participants were briefed about the research topic, its aim and the types of questions. They were also informed about the confidentiality of the data being collected, and no personal information or identification was retained, with each interviewee being assigned a unique number. Interviewees working in the public sector expressed their reluctance to sign consent forms; therefore, verbal consent was adopted for this study and obtained from each participant prior to the interview. All procedures performed in studies involving human participants were in accordance with the ethical standards of the institutional and/or national research committee and with the 1964 Helsinki Declaration and its later amendments or comparable ethical standards.

## Results

The resilience indicators identified under the 3 components of the CIERA method are provided in Tables 2, 3 and 4. While some indicators are similar to those obtained in the literature review for other CI systems, especially when looking at those pertaining to organisational resilience, several others have emerged from the thematic analysis that are more specific to the road infrastructure sector and its technical aspects.

**TABLE 2:** Development of indicators for the robustness component.

Interview transcripts	Codes	Indicators	Variables
‘… need coordination between stakeholders using a common platform to ensure that everyone is aware of potential risks and problem areas’.	Aware of potential risks and problem areas	Level of awareness of risk prone areas	Crisis preparedness
‘… to share land maps of flood prone areas to allow planning for resiliency and carry out impact assessments in terms of traffic and drainage’.	Share land maps of flood prone areas		
‘… have staff at regional level to intervene after the emergency situations’.	Staff to intervene after disasters	Dedicated post-disaster intervention team	
‘… have a dedicated team to carry out immediate inspection after events …’	Dedicated team for inspection		
‘… include risk analysis when studying different road alignment alternatives …’	Risk analysis	Preparedness level through risk audit	
‘… obtain inundation maps from authorities to assess risks before implanting projects …’	Assess risks		
‘… should not only consider basic aspects when designing … but also carry out a risk audit …’	Risk audit		
‘… aim to have grid patterns in the road network to have alternative routes if one part is closed’.	Alternative routes	Availability of alternative routes	Redundancy
‘At several places, bypass roads provide an alternative link in case one road is affected’.	Alternative link		
‘… need a framework for agencies to work during disasters and maintain a basic level of operations’.	Maintain basic level of operations	Business continuity of institution	
‘… need to have redundancy in people performing different tasks to ensure that work can continue …’	Ensure work can continue		
‘Warning systems should be able to detect and report early on potential risks so that planned measures can be put in place …’	Warning systems able to detect and report early	Early warning systems with detection and reporting abilities	Detection capability
‘… prevent lifespan to be reduced for example through early warning on pavement distress …’	Early warning		
‘… planned inspection routines of assets to prevent excessive deterioration of pavements …’	Inspection of assets	Asset monitoring capabilities	
‘… regular monitoring of assets to identify potential risks of flooding due to blocked drains’.	Monitoring of assets		
‘… drain network should be monitored and cleaned systematically before the rainy season’.	Monitored and cleaned		
‘… there should be established plans of most affected areas for planning works …’	Plans of most affected areas	Ability to analyse and map affected areas	
‘Obtaining data is essential to allow engineers to analyse areas and have a priority list of projects’.	Analyse areas and have a priority list		
‘… team and equipment should be available so that little time is taken to address small issues …’	Little time to address issues	Recovery time	Responsiveness
‘… provide adequate information and resources to have quick recovery on affected parts …’	Quick recovery		
‘Real time information should be given to people on affected roads and evacuation routes …’	Real time information	Information systems to manage road users	
‘… have information being provided to road users through systems such as message alerts’.	Information systems		
‘… use worst case scenarios to design protective measures such as drains and culverts …’	Protective measures	Presence of protective measures	Physical resistance
‘… identify areas with soil stabilization measures and implement measures to protect the road infrastructure such as nailing …’	Implement measures to protect roads		
‘… adopt codes which cater for design with climate change considerations …’	Codes for design	Adoption of design standards incorporating climate change	
‘… select appropriate design criteria for more resilience such as greater return periods …’	Appropriate design criteria		

**TABLE 3:** Development of indicators for the recoverability component.

Interview transcripts	Codes	Indicators	Variables
‘… for the supply of materials, contractors need to make use of local materials which are readily available to reduce time of repairs’.	Materials which are available	Availability of materials for repairs	Material resources
‘… there should be enough materials available immediately to carry out repair works’.	Materials available		
‘… need to also consider how easy it will be to repair the elements after a disaster’.	Easy to repair the elements	Repair potential of element	
‘… have materials which are more resilient and locally available to avoid delays in repairs’.	Locally available		
‘… but to be able to plan for resilient systems, we need to allocate a budget for resilience especially in emergency situations’.	Allocate budget for emergency situations	Availability of funding for resilience	Financial resources
‘… unwillingness to invest in resilience due to the high costs and limited budget …’	High costs and limited budget		
‘… established procedures need to be clearly set and communicated for action to be taken in a timely manner as huge funds may be involved’.	Established procedures for action	Established emergency procurement process	
‘Generally, the procurement process is very rigid but there is a need to have agreed procedures on how to act during emergency …’	Agreed procedures for emergency		
‘… train personnel to use special equipment and have them available during disasters’	Personnel availability	Availability of human resources	Human resources
‘… need expert personnel and trained labour to be available during disasters …’	Available during disasters		
‘… limited technical ability of staff prevents adoption of new adaptation strategies …’	Technical ability of staff	Technical expertise of human resources	
‘… provide training to staff to increase their knowledge on resilience measures …’	Increase knowledge		
‘… response plans have to be developed and approved especially for critical locations …’	Plans developed and approved	Pre-approved response plans	Recovery processes
‘… should not wait for the disaster to happen to come up with a response plan …’	Response plan		
‘… need to have agreements with foreign countries who have expertise in disaster …’	Agreement with foreign experts	Agreement with third parties for support	
‘… have agreement with local contractors who have the capacity to work in emergencies’.	Agreement with contractors		

**TABLE 4:** Development of indicators for the adaptability component.

Interview transcripts	Codes	Indicators	Variables
‘… have a risk management system to ensure that issues have already been identified …’	Risk management system	Level of risk assessment methods	Risk management
‘… a risk assessment methodology when designing or constructing …’	Risk assessment methodology		
‘… need to conduct risk analysis to determine problem areas and solutions …’	Risk analysis		
‘… should have the capacity to carry out risk analysis and devise maintenance plans …’	Capacity to carry out risk analysis	Risk management capacity	
‘… need to be able to develop models … during disasters to devise risk management plans’.	Risk management plans		
‘… need to develop simulation exercises in case of disasters based on maps of critical areas’.	Simulation exercises	Scenario development level for disruptions	
‘… simulations to develop evacuation plans based on different critical scenarios …’	Simulations for scenarios		
‘… should be able to model the road infrastructure to identify actual security standards and measures to improve …’	Actual security standards and measures	Implementation of security standards	
‘…need to identify on maps places with low security levels and potential measures to be implemented to increase the standards …’	Increase the security standards		
‘… require more research on climate resilient materials to develop new specifications …’	More research on resilient materials	Research and development	Innovation processes
‘… need to increase studies on materials to develop more resilient ones …’	Increase studies on materials		
‘… allow for bidding procedures such as design-and-build which allow contractors to propose more resilient alternatives …’	Innovative bidding procedures	Innovative procurement process	
‘… provide incentives and assessment criteria in the bidding documents for contractors proposing measures favouring resilience …’	Incentives in procurement process		
‘…adopt innovative asphalt and concrete adapted to local context and use of resilient materials for road furniture also’.	Adopt innovative materials	Technology innovation	
‘… need to have new technology to be able to have new construction possibilities …’	New technology for construction		
‘… develop maintenance and operation manuals for management of before and after disasters …’	Manuals for management	Implementation of management systems	
‘… use of asset management and databases to establish priorities for interventions …’	Asset management		
‘… measures for resiliency tend to be costly and there is a need to increase the level of investment in use of innovative materials and construction technologies’.	Increase level of investment	Innovation investment level	
‘… there is no dedicated budget for resiliency or innovation in projects …’	Dedicated budget		
‘…local staff do not have the expertise to deal with critical events and should be provided with training …’	Provided with training	Provision of training to deal with disruptions	Education and development processes
‘… limited understanding of resilience and how to incorporate it in road projects, with training workshops important to make them aware of climate change adaptation measures’.	Training workshops		
‘… should not only attend training sessions but should also be evaluated on the effectiveness of the materials followed using real case scenarios’.	Evaluated on the effectiveness of materials	Evaluation of training effectiveness	
‘… undergo training on modelling tools followed by an evaluation to ensure people can successfully use the tools when working’.	Evaluation … use tools		
‘… promote sharing of knowledge for everyone to learn from past events …’	Promote sharing … to learn	Proactive work and learning culture	
‘… need to have the participation of everyone to discuss new ideas, learn new techniques and embrace change’.	Learn new techniques		
‘… instill proactive working conditions among staff to overcome resistance to change …’	Proactive working conditions		

### Robustness component

Robustness referring to the ability of an element to absorb the impacts of a disruption comprises five components for which 12 indicators were obtained as shown in Table 2.

### Recoverability component

For the recoverability aspect which refers to the capacity of an element to recover its function to the required level after a disruptive event, eight indicators emerged for the four variables as detailed in Table 3.

### Adaptability component

Adaptability refers to the ability of an operator to prepare an element for the potential effects of disruptive events, and 12 indicators were obtained for the three variables of this component as detailed in Table 4.

## Discussion

**FIGURE 2:**
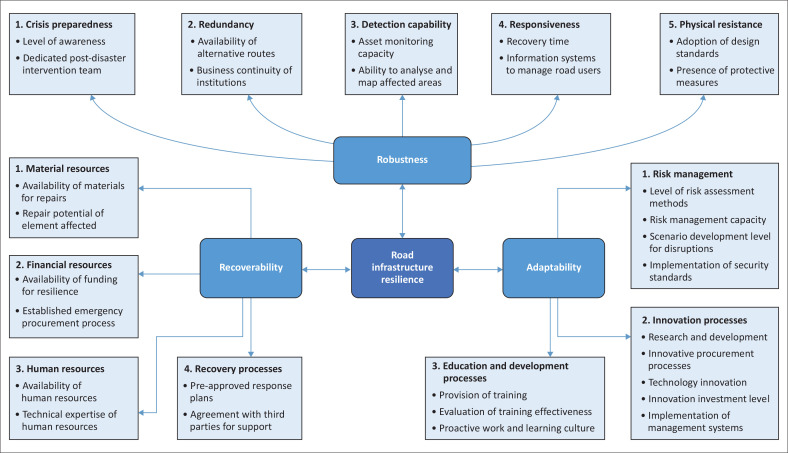
Critical infrastructure elements resilience assessment framework with components and resilience indicators for road infrastructure.

The primary purpose of this study was to identify important indicators for inclusion in the CIERA method adapted to assess road infrastructure resilience. Several of the indicators obtained are consistent with previous studies on resilience to disaster events. Pathirage et al. (2014) identified innovative technological tools for early warning, communication systems for mass communication and structural measures as important factors together with financial policies and adequate risk management skills and knowledge. The need for training programmes as part of capacity building of organisations, provision of dedicated funds for emergency situations, the need to build infrastructure redundancy and carry out designs for reduced deterioration have also been highlighted (Weilant, Aaron & Benjamin et al. 2019), while the availability of several connections to an area is also deemed essential for improved resilience (Serre & Heinzlef 2018). On the organisational aspect, previous studies identified risk management capabilities and innovation adoption as essential for improved resilience (Rehak et al. 2019). A total of 32 indicators were obtained for the three components of robustness, recoverability and adaptability, with the corresponding variables and components shown in the framework in Figure 2.

Based on the indicators obtained, the following practical recommendations are suggested for organisations in the road sector:

In terms of robustness, the improvement of the physical resistance of the road infrastructure will inevitably require the adoption of stringent design standards that cater for climate change and more conservative design criteria for stormwater systems and road pavements. The implementation of protective measures, especially in areas prone to landslide in terms of soil stabilisation, nailing and construction of retaining walls, is also essential for increased resistance to flood events. For greater crisis preparedness, the sharing of available data and maps among stakeholders on a common platform has been proposed to improve the level of awareness of critical sites, while having dedicated post-disaster intervention teams will also allow recovery measures to be taken quicker. Construction of alternative routes in high-risk locations and ensuring that institutions continue to operate at satisfactory service levels have also emerged as important measures for redundancy in the system. For detection capability, setting up of a proper asset monitoring system will help to prevent excessive deterioration of infrastructure by allowing early identification of problems while training personnel on the analysis and mapping of critical locations will ensure that proper measures are taken in priority areas. In terms of responsiveness, reducing the time for recovery to normal operations through quick repairs and having real-time information systems to guide road users, especially during evacuation through variable message signs on roads or message alerts on mobile phones, are potential measures that could be implemented.

For the recoverability component, it is essential to provide financial resources needed for works to be carried out in emergency situations and to implement resilience in road projects, as this usually entails higher costs. Development of pre-approved response plans and entering into agreement with third parties such as local contractors and foreign experts in disaster recovery will also help to ensure that recovery processes after a disaster are well established. For works to be carried out after a disaster to restore the infrastructure, there is inevitably the need for institutions to have adequate material and well-trained human resources to undertake repairs quickly.

Lastly, for the adaptability component, participants were of the view that overcoming resistance to change and embracing innovation is essential to move towards greater resilience in the road infrastructure. Thus, research on new materials and development of specifications for these, engaging in innovative procurement processes with incentives for resilience and investing in new technologies are a few of the essential measures that should be taken by institutions. In terms of risk management, building risk management capacity and risk assessment methodologies are essential at organisation level, while on the technical side, the ability to carry out modelling of various disruption scenarios and implement security standards is essential for improved resilience. Finally, most interviewees concluded that knowledge on resilience is still lacking at all levels, with organisations fostering a proactive work and learning culture necessary to overcome this issue. Moreover, adequate training should be provided and supplemented by evaluation exercises to assess its effectiveness.

## Conclusion

Road transport infrastructure is an essential component of CI systems as it is the backbone for movement of people, economic activities and emergency services. Incorporating resilience in road networks is therefore essential to ensure that they continue to perform at satisfactory levels during and after disruption events. Although several studies have been carried out to study resilience of roads, these have been largely focused on mathematical modelling and simulation at network level, with a void on performance indicators to measure resilience at element level. This study was conducted to identify the indicators for measuring road infrastructure elements resilience using the CIERA framework, which has already been applied in other CI domains such as energy and power systems. The results of this qualitative study revealed 32 major indicators under the three main components of robustness, recoverability and adaptability for the technical and organisational dimensions. Transportation professionals can use these indicators to assess the level of resilience of elements in the road network and thus determine which are the most vulnerable. This method will help to better allocate limited funds and resources for improving resilience in the road network and hence improve capacity to withstand the adverse effects of disruptive events.

However, the study had some limitations as a quantitative approach was not undertaken to determine ranking and weightages of the indicators. Future research should therefore aim to carry out a survey with professionals working in the transport infrastructure sector to determine the weightages associated with each indicator. Moreover, future studies can consider extending the proposed CIERA method for road infrastructure with other components such as resistance, resourcefulness and rapidity or other dimensions such as social, economic and environmental.
